# Commentary: Evaluation of the AiDx Assist device for automated detection of *Schistosoma* eggs in stool and urine samples in Nigeria

**DOI:** 10.3389/fpara.2025.1633767

**Published:** 2025-07-22

**Authors:** Nathkapach Kaewpitoon Rattanapitoon, Schawanya Kaewpitoon Rattanapitoon

**Affiliations:** Parasitic Diseases Research, FMC Medical Center of Thailand, Nakhonratchasima, Thailand

**Keywords:** AI-powered diagnostics, schistosomiasis, AiDx Assist evaluation, stool samples, urine samples

The recent article by Meulah et al. (2025) represents a commendable step toward realizing AI-integrated microscopy as a scalable diagnostic solution for schistosomiasis. The validation of AiDx Assist in a dual-endemic setting for *S. haematobium* and *S. mansoni* reflects a well-designed response to the WHO’s call for point-of-care tools meeting target product profiles (World Health Organization, 2021). Particularly notable is the strong sensitivity and specificity (>90%) achieved in detecting *S. haematobium* in urine, both in semi-automated and fully automated modes. These results suggest readiness for deployment in urogenital schistosomiasis control programs.

However, the relatively lower sensitivity of the fully automated detection for *S. mansoni* in stool (56.9%) warrants further algorithm refinement. The discrepancy between semi- and fully automated performance suggests that AI misclassification or under-detection remains a technical bottleneck—likely influenced by the morphological variability and background complexity of stool slides (Bogoch et al., 2013; Coulibaly et al., 2016). One avenue to improve performance could be the integration of convolutional neural networks trained on a broader dataset including diverse egg presentations and artifacts (McManus et al., 2018).

A notable strength of the study is its dual-sample analysis (stool and urine) in a field setting—a rare approach that mimics real-world application. Moreover, the incidental visualization of *Ascaris lumbricoides* and *Trichuris trichiura* eggs in retrospect highlights the potential of AiDx Assist as a multi-parasite detection platform. We propose formalizing this potential through a prospective multi-pathogen training dataset and validation study, as demonstrated by other AI-parasitology platforms (Hemachandran et al., 2023; Kittur et al., 2022).

To further bolster the impact and utility of AiDx Assist, we suggest three enhancements:

1. Expand stool slide training sets to include polyparasitism and low-intensity infections, thus aligning performance with the WHO-recommended Kato–Katz sensitivity thresholds.
2. Develop modular AI plug-ins for soil-transmitted helminths, aligning with WHO’s integrated helminth control strategies dating back to early guidance (World Health Organization, 2002) and reaffirmed in the 2030 NTD roadmap (World Health Organization, 2021).
3. Pilot longitudinal field evaluations to assess device durability, technician learning curves, and integration into MDA programs.

If these are pursued, AiDx Assist could evolve into a truly transformative tool—not only for schistosomiasis control but for broader parasitic diagnostics in LMICs.
